# Small airway epithelial-C/EBPβ is increased in patients with advanced COPD

**DOI:** 10.1186/s12931-015-0297-0

**Published:** 2015-10-29

**Authors:** Michiko Mori, Leif Bjermer, Jonas S. Erjefält, Martin R. Stampfli, Abraham B. Roos

**Affiliations:** Department of Experimental Medical Science, Lund University, Lund, Sweden; Department of Respiratory Medicine and Allergology, Lund University, Lund, Sweden; Department of Pathology and Molecular Medicine, McMaster University, MDCL 4084, 1280 Main Street West, Hamilton, ON L8S 4P1, Canada; Department of Medicine, Firestone Institute of Respiratory Health at St. Joseph’s Health Care, Hamilton, ON Canada

**Keywords:** COPD, C/EBPβ, Airway epithelium

## Abstract

The expression of CCAAT/enhancer-binding protein (C/EBP)β in the small airway epithelium of COPD is unknown. C/EBPβ was assessed in peripheral lung tissue of non-smoking/smoking controls and patients with GOLD I-IV COPD by quantitative immunohistochemistry. The expression of C/EBPβ was decreased in smokers compared to never smokers. Furthermore, C/EBPβ was significantly elevated in advanced COPD vs. asymptomatic smokers, and the expression correlated to lung function decline. As C/EBPβ exerts pro-inflammatory effects in the context of cigarette smoke, the elevated C/EBPβ in advanced COPD may be an indication of a breakdown of regulatory mechanisms and excessive inflammation.

## Findings

Chronic obstructive pulmonary disease (COPD) is characterized by small airway inflammation. While glucocorticoids (GCs) and β_2_ agonists are mainstay in the management of COPD, these classes of drugs are, by and large, ineffective in preventing disease progression [1]. The lack of efficient pharmaceutical options is in part due to the incomplete understanding of the intricate molecular mechanisms contributing to the disease.

The transcription factor CCAAT/enhancer binding protein (C/EBP)β regulates inflammatory [2] and host defense genes [3] in the airway epithelium. Lung epithelial-C/EBPβ activates the inflammatory response to cigarette smoke [4], as well as lipopolysaccharide (LPS) [3]. Suppression of LPS-induced airway inflammation by β_2_ agonists is, however, also mediated by lung epithelial-C/EBPβ [3]. In addition, glucocorticoids increase the expression and transcriptional activity of C/EBPβ. Transactivation by glucocorticoids has in contrast to pro-inflammatory stimuli been suggested to up-regulate host defense genes [5, 6]. Hence, cigarette smoke and microbial ligands, as well as GCs and β_2_ agonists may all activate airway-epithelial C/EBPβ in COPD, with the possibility of different outcomes depending on the stimuli. There is currently insufficient knowledge of the expression of C/EBPβ in the small airways of COPD, in particular in end-stage disease where GC/β_2_ agonist therapy is mainstay.

We obtained peripheral tissue specimens from patients with stable GOLD I-IV COPD (*n* = 30), as well as controls with or without a smoking history (*n* = 14) (Table 1). The study was approved by the Swedish Research Ethics Committee in Lund, Sweden. All study subjects signed informed consent to participate. Formalin-fixed and paraffin-embedded tissue sections were pre-treated with a pH 6.1 buffer (EnVision™ FLEX Target Retrieval Solution, Dako, Glostrup, Denmark). The expression of C/EBPβ was visualized by immunohistochemistry using a polyclonal rabbit anti-C/EBPβ antibody (Santa Cruz Biotechnology, Santa Cruz, CA, USA), and EnVision™ Peroxidase/DAB Detection System kit on an Autostainer Plus (DakoCytomation, Glostrup). Automated immunohistochemistry allowed for minimized operator error between tissue samples.

**Table 1:** Baseline demographics and clinical characteristics

Parameter	Never smokers	Smokers w/o COPD	GOLD I-II COPD^b^	GOLD III-IV COPD^c^	*p* ANOVA
Subjects (n)^a^	8	6	18	12	
Gender (female/male)	6/2	3/3	5/13	6/6	
Age (years)	63 ± 4.8	56 ± 3.2	68 ± 1.8	61 ± 1.2	<0.05
Height (m)	1,64 ± 0.033	1.72 ± 0.05	1,74 ± 0.02	1,7 ± 0.031	ns
Weight (kg)	64,6 ± 4.6	69.2 ± 4.4	73,1 ± 3.5	67,7 ± 4.1	ns
Body mass index	23,9 ± 1.3	23.3 ± 1.1	24,4 ± 1.1	23,3 ± 0.94	ns
Pack years	N/A	43 ± 9.7	45 ± 3.5	41 ± 3.2	ns
Smoker/ex-smoker	N/A	3/3	7/11	0/12	
FEV1/FVC	85,9 ± 5.7	77.8 ± 2.4	61,4 ± 1.9	33,4 ± 2.1	<0.001
FEV1 (% of predicted)	109,8 ± 6.2	93.8 ± 4.2	74,1 ± 2.7	26,2 ± 2.7	<0.001
Corticosteroids (yes/no/unknown)	0/8/0	0/6/0	2/16/0	9/2/1	
Bronchodialator (yes/no/unknown)	0/8/0	0/6/0	6/12/0	9/2/1	

^a^All surgeries were performed at Skåne University Hospital, in Lund, Sweden

^b^Tissue samples were obtained during lung resection surgery for bronchial tumour

^c^Tissue samples were obtained from GOLD II COPD patients during lung resection surgery for bronchial tumour, and from GOLD IV COPD patients during lung transplantation

### C/EBPβ is decreased in the small airway epithelium of asymptomatic smokers

**Fig. 1:**
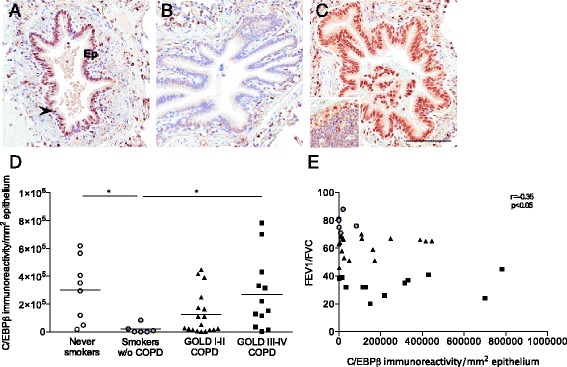
Reduced expression of C/EBPβ in asymptomatic smokers and elevated expression in advanced COPD. Light micrographs of the immunoreactivity to CCAAT/enhancer-binding protein (C/EBP)β in peripheral pulmonary tissue of a (**a**) never-smoker, (**b**) asymptomatic smoker, and (**c**) patient with very severe chronic obstructive pulmonary disease (COPD). Immunoreactivity was detected by DAB (brown). Sections were counterstained with Mayer’s Hematoxylin (blue). Scale bar indicates 100 μm. The epithelium (Ep) is denoted and an arrowhead indicates a positive cell in (**a**). Inlet of (**c**) shows immunoreactivity to C/EBPβ in a tertiary lymphoid follicle. **d** Immunoreactivity (defined as number of positive pixels/mm^2^) to C/EBPβ in peripheral lung epithelium of never-smokers, asymptomatic smokers and patients with mild-moderate (global initiative of COPD, GOLD I-II) and severe-very severe (GOLD III-IV) COPD. Horizontal lines indicate mean value. **e** Pearson correlation coefficient analysis of the immunoreactivity/mm^2^ to C/EBPβ in peripheral lung epithelium and the forced expiratory volume in 1 second (FEV1)/forced vital capacity (FVC) of smokers without COPD and patients with GOLD I-IV COPD. Clear circles: never smokers without airway obstruction; grey circles: asymptomatic smokers, triangles: GOLD I-II COPD; squares: GOLD III-IV COPD. *n* = 6-18. *p < 0.05

Strong immunoreactivity to C/EBPβ was observed in the peripheral airway epithelium, as well as in alveolar macrophages of COPD patients and asymptomatic controls (Fig. 1a-c). C/EBPβ positive cells were furthermore identified within and in the epithelial interface of lymphoid follicles [7], in lung tissue collected from patients with COPD (inlet of Fig. 1c).

An Aperio ScanScope Slide Scanner (Aperio Technologies, Vista, CA) was used to generate digital images of the tissue sections, and morphometric analyses were performed using Aperio ImageScope v.10.0 software (Aperio Technologies) [8]. Computerized image analysis revealed that the expression of C/EBPβ was significantly lower in the airway epithelium among asymptomatic controls with a smoking history compared to never smokers (*p* < 0.05, Fig. 1d). The lower expression of C/EBPβ was associated with a reduced immunoreactivity to the Marker of Proliferation (M) KI67 (rabbit polyclonal antibody A0047, DakoCytomation) suggestive of an attenuated proliferation of the airway epithelium (0.02 ± 0.004 vs. 0.0082 ± 0.0026; mean ± SEM, *p* < 0.01).

The reduced expression of C/EBPβ corroborates our previous finding of significantly decreased *CEBPB* mRNA in the bronchial epithelium of current and former smokers, compared to never smokers [4], and reduction of *CEBPB* mRNA and C/EBPβ protein in bronchial epithelial cells stimulated with cigarette smoke extract *in vitro* [4]. Thus, C/EBPβ is down-regulated by cigarette smoke in both the proximal and distal airway epithelium. This may be part of a compensatory mechanism of feed back inhibition, as an adaptive attempt to control chronic inflammation. C/EBPβ contributes to the differentiation of the airway epithelium during organogenesis, and promotes club cell differentiation at the expense of goblet cell differentiation [9]. As cigarette smoke stimulates goblet cell differentiation *in vitro* [10], decreased expression of C/EBPβ in the distal airways may thus provide a mechanistic explanation for goblet cell hyperplasia induced by cigarette smoke. While the smokers included in our study were asymptomatic, decreased C/EBPβ may over time lead to clinical presentation with mucus hypersecretion. In support of this, the activity of C/EBPβ in the bronchial epithelium is decreased in smokers with chronic bronchitis [11], compared to asymptomatic smokers.

### Airway epithelial-C/EBPβ is elevated in advanced COPD

The expression of airway epithelial-C/EBPβ was significantly increased in advanced (GOLD III-IV) COPD, compared to asymptomatic smokers (*p* < 0.05, Fig. 1d). Furthermore, a negative correlation between lung function and the airway expression of C/EBPβ was observed (*r* = −0.35 *p* < 0.05, Fig. 1e), suggesting a role for C/EBPβ in disease progression. The expression of the lung-enriched C/EBPα, which cooperates with C/EBPβ in various cellular functions [2], was not significantly different in any of the groups within the cohort (C/EBPα rabbit polyclonal antibody (14AA) sc-61, Santa Cruz Biotechnology, Dallas, TX, USA; data not shown).

Mechanistically, it is possible that the elevation of C/EBPβ represents a breakdown of the suggested feed back inhibition observed in cigarette smoke-induced inflammation, leading to escalating inflammatory processes in end-stage COPD. Alternatively, chronic bacterial colonization among COPD patients [12] may activate C/EBPβ. It is, however, also possible that steroid and β_2_ agonist treatment effected the expression of C/EBPβ in our study, as airway epithelial-C/EBPβ is induced/activated by GCs and β_2_ agonists [3, 6]. This may represent a novel mechanism by which GCs and β2 agonists modulate the transcriptional profile of the airway epithelium in advanced COPD. Future studies should address whether the elevation of C/EBPβ is disease- or treatment-specific, and if GCs and β_2_ agonists induces C/EBPβ to promote host-defenses and act anti-inflammatory, or pro-inflammatory.

## References

[1] Vestbo J, Hurd SS, Agustí AG, Jones PW, Vogelmeier C, Anzueto A, Barnes PJ, Fabbri LM, Martinez FJ, Nishimura M (2013). Global Strategy for the Diagnosis, Management, and Prevention of Chronic Obstructive Pulmonary Disease. Am J Respir Crit Care Med.

[2] Cassel TN, Nord M (2003). C/EBP transcription factors in the lung epithelium. Am J Physiol Lung Cell Mol Physiol.

[3] Roos AB, Barton JL, Miller-Larsson A, Dahlberg B, Berg T, Didon L, Nord M (2012). Lung epithelial-C/EBPbeta contributes to LPS-induced inflammation and its suppression by formoterol. Biochem Biophys Res Commun.

[4] Didon L, Barton JL, Roos AB, Gaschler GJ, Bauer CM, Berg T, Stampfli MR, Nord M (2011). Lung epithelial CCAAT/enhancer-binding protein-beta is necessary for the integrity of inflammatory responses to cigarette smoke. Am J Respir Crit Care Med.

[5] Zhang N, Truong-Tran QA, Tancowny B, Harris KE, Schleimer RP (2007). Glucocorticoids Enhance or Spare Innate Immunity: Effects in Airway Epithelium Are Mediated by CCAAT/Enhancer Binding Proteins. J Immunol.

[6] Berg T, Cassel TN, Schwarze PE, Nord M (2002). Glucocorticoids regulate the CCSP and CYP2B1 promoters via C/EBPbeta and delta in lung cells. Biochem Biophys Res Commun.

[7] Mori M, Andersson CK, Svedberg KA, Glader P, Bergqvist A, Shikhagaie M, Löfdahl C-G, Erjefält JS (2013). Appearance of remodelled and dendritic cell-rich alveolar-lymphoid interfaces provides a structural basis for increased alveolar antigen uptake in chronic obstructive pulmonary disease. Thorax.

[8] Roos AB, Mori M, Gronneberg R, Osterlund C, Claesson HE, Wahlstrom J, Grunewald J, Eklund A, Erjefalt JS, Lundberg JO, Nord M (2014). Elevated exhaled nitric oxide in allergen-provoked asthma is associated with airway epithelial iNOS. PLoS One.

[9] Roos AB, Berg T, Barton JL, Didon L, Nord M (2012). Airway epithelial cell differentiation during lung organogenesis requires C/EBPalpha and C/EBPbeta. Dev Dyn.

[10] Haswell LE, Hewitt K, Thorne D, Richter A, Gaça MD (2010). Cigarette smoke total particulate matter increases mucous secreting cell numbers in vitro: A potential model of goblet cell hyperplasia. Toxicol In Vitro.

[11] Didon L, Qvarfordt I, Andersson O, Nord M, Riise GC (2005). Decreased CCAAT/Enhancer Binding Protein Transcription Factor Activity in Chronic Bronchitis and COPD. Chest.

[12] Murphy TF, Brauer AL, Schiffmacher AT, Sethi S (2004). Persistent colonization by Haemophilus influenzae in chronic obstructive pulmonary disease. Am J Respir Crit Care Med.

